# Sex workers' professional agency, quality of life, and problematic substance use in Finland

**DOI:** 10.1111/sjop.13070

**Published:** 2024-09-15

**Authors:** Annika Gunst, Mimmi Uusitalo, Petra Pölönen, Štefan Petrík, Jan Antfolk

**Affiliations:** ^1^ Department of Psychology Åbo Akademi University Turku Finland; ^2^ National Institute of Education and Youth Banská Bystrica Slovakia

**Keywords:** Sex work, professional agency, quality of life, well‐being, substance use, alcohol

## Abstract

**Introduction:**

Sex workers suffer considerable marginalization that limits their choices and exposes them to various types of harm. Hence, it is important to examine sex workers' professional agency and its association with quality of life. In the current study, we investigated professional agency, quality of life, and problematic substance use among sex workers in Finland.

**Methods:**

Using an online survey, we collected data from 136 sex workers contacted through Finnish sex work organizations and social media platforms. We conducted correlational analyses between the study variables and linear regression analyses with professional agency as the independent variable and quality of life and problematic alcohol and drug use as dependent variables.

**Results:**

As expected, in the regression analyses, professional agency was strongly positively associated with quality of life (β = 0.86, *p* < 0.001) and negatively associated with problematic alcohol (β = −0.38, *p* = 0.002) and drug (β = −0.69, *p* < 0.001) use. Professional agency explained as much as 73% of the variance in quality of life. Most sex workers rated their quality of life as either good or very good.

**Conclusion:**

Our results underscore the importance of professional agency to sex workers' well‐being, explaining a substantial proportion of the variance in quality of life. Promoting agency should therefore be at the heart of sex work policymaking. However, as most of the respondents were Finnish‐born cis women, our results might not generalize to foreign‐born, male, and gender diverse sex workers. Future studies should prioritize reaching these populations to ensure broader representativeness.

## INTRODUCTION

Sex workers are often considered one of the world's most marginalized and stigmatized groups (Amnesty International, 2016). Sex work has traditionally been defined as selling sex in exchange for money, economic benefits (e.g., holiday trips), or immediate needs (e.g., food; Krumrei‐Mancuso, 2017; Liu, Srikrishnan, Zelaya, Solomon, Celentano & Sherman, 2011; Wolf, 2019). In the scientific literature, most studies on sex workers have focused on street‐based sex workers providing physical services (Ham & Gerard, 2014). However, the incessant and recent changes in the sex work industry, such as the increased promotion of sex work online during the past decade (Selvey, Hallett, Lobo, Mccausland, Bates & Donovan, 2017), warrant a broader definition of sex work that includes services that do not involve sexual or physical contact. Such non‐physical services can include media‐based services (e.g., *OnlyFans*, webcamming, and pornography) and services provided in person (e.g., escort services, dance, stripping, and girl‐ or boyfriend experiences).

It has been estimated that there are between 5,000 and 6,000 sex workers in Finland and that most of the sex workers are women (90%), are migrants (69%), and work indoors (90%; TAMPEP, 2010). These numbers are indicative, as they were given more than a decade ago and do not take media‐based sex workers into account. The number of sex workers in Finland is also likely underestimated as most of their work remains invisible (Kontula, 2008).

### Sex work and quality of life

To date, there are only a few quantitative studies on the quality of life of sex workers. In an Indian study, the authors concluded that the quality of life of sex workers was generally poor (Shukla & Mehrotra, 2014). In two Spanish studies using the same sample of sex workers, the authors found that loneliness, violence, and drug use were associated with poorer mental health, which in turn was associated with lower quality of life (Picos, González & de la Iglesia Gutiérrez, 2018; Pinedo González, Palacios Picos & de La Iglesia Gutiérrez, 2021). In a Brazilian study, more than half of the participants reported signs of mental health disorders (Elias, de Araújo & de B. Junqueira, 2020). A Dutch study found that sex workers, on average, scored just slightly below the cutoff for clinical depression (Krumrei‐Mancuso, 2017). Experiences of violence, street‐based sex work, financial motives, a desire to leave the profession, and a lower sense of agency were associated with elevated mental health concerns. A study from southern China that included more than 1,000 female sex workers found that alcohol use was associated with more mental health problems (Zhang, Li, Chen, *et al*., 2014). In an Iranian study, the authors found that drug use was associated with decreased quality of life (Khodabakhshi Koolaee & Damirchi, 2016).

It is legal to provide and buy sex in Finland (Platt, Grenfell, Meiksin, *et al*., 2018; Pro‐tukipiste, 2021). Laws and restrictions nonetheless regulate sex work: It is, for instance, illegal to offer and buy sexual services in public spaces and to buy sexual services if the person who provides sexual services is underage or a victim of sex trafficking (Pro‐tukipiste, 2021). An alien may also be subject to deportation if there are reasonable grounds to suspect that they may sell sexual services (Pro‐tukipiste, 2021). Due to differences in laws and restrictions between countries, research findings outside Finland might not generalize to Finnish conditions. For example, a meta‐analysis showed that adverse health outcomes were more common in jurisdictions that had criminalized sex work (Platt, Grenfell, Meiksin, *et al*., 2018). Prior studies on sex workers in Finland have mainly used qualitative designs with small samples (e.g., Diatlova, 2019; Kontula, 2008; Skaffari, 2010; Tähtinen, 2021). In the only quantitative study investigating sex workers' health and well‐being, the authors found that about half of their participants were either satisfied or very satisfied with their lives (Liitsola, Kauppinen, Nikula, *et al*., 2013). Additional research is needed to increase understanding of potential health concerns and needs for health services among sex workers in Finland (Regushevskaya, Haavio‐Mannila, Tuormaa & Hemminki, 2017).

### Professional agency and quality of life

Professional agency is conceptualized as control to make choices and take stances on one's work and professional identity (Eteläpelto, Vähäsantanen, Hökkä & Paloniemi, 2013). Studies in other professions suggest that a higher professional agency is associated with a better quality of life (for a meta‐analysis, see Ng, Sorensen & Eby, 2006). Professional agency has previously been discussed in relation to sex work, and the discussion has traditionally divided researchers. Some argue that entry into sex work results from a poor financial situation and that sex workers are, therefore, victims without agency. In contrast, others argue that this argument can also be applied to any type of work (Bungay, Halpin, Atchison & Johnston, 2010). More recent studies have suggested that this polarized discussion is a simplification, that many factors impact sex workers' agency, and that agency comes in degrees (Bettio, Della Giusta & Di Tommaso, 2017). Moreover, many sex workers do not view themselves as victims (Sagar & Jones, 2014). However, given that sex workers are structurally vulnerable, which is assumed to limit a person's choices and make them more likely to be exposed to various types of harm (Footer, White, Park, Decker, Lutnick & Sherman, 2020; Nestadt, Tomko, Schneider, Kerrigan, Decker & Sherman, 2020), it is relevant to examine sex workers' professional agency and how it is associated with their quality of life.

Some studies have found that sex workers' agency is negatively associated with, for instance, severe mental illness (Buttram, Surratt & Kurtz, 2014), alcohol use (Levi‐Minzi, Surratt, O'Grady & Kurtz, 2016), drug use, violence, and homelessness (Nestadt, Tomko, Schneider, Kerrigan, Decker & Sherman, 2020). One of the reasons for providing sexual services may be to acquire drugs (e.g., Sherman, Park, Galai, *et al*., 2019). In such cases, the sex worker's sense of agency can be restrained due to their drug addiction and their need to acquire drugs, which, in turn, may lead to more unprotected sex (Ditmore, 2013; Nestadt, Tomko, Schneider, Kerrigan, Decker & Sherman, 2020; Strathdee, Philbin, Semple, *et al*., 2008). In contrast, sex workers' agency has been positively associated with physical health (Dalla, Xia & Kennedy, 2003) and social support (Buttram, Surratt & Kurtz, 2014).

### The current study

In the current study, we investigated how the professional agency of sex workers residing in Finland is associated with their quality of life and problematic substance use. To our knowledge, this is the first quantitative study to investigate sex workers' professional agency in Finland and the first study in Finland to include media‐based sex workers. Based on previous research, we derived the following hypotheses:

Hypothesis 1: Sex workers' professional agency will be positively correlated with quality of life.

Hypothesis 2: Sex workers' professional agency will be negatively correlated with problematic substance use.

We also explored how different demographic aspects (i.e., financial situation, educational level) correlated with professional agency, quality of life, and problematic substance use.

## MATERIALS AND METHODS

### Ethical permission

The Board for Research Ethics at Åbo Akademi University granted ethical approval for the present study on February 7, 2022, before the start of our data collection.

### Respondents

Participation required the sex worker to be at least 18 years old and to have been providing sexual services in Finland for at least the past 6 months. Of the 155 who started the survey, 99 respondents completed it, resulting in a completion rate of 63.9%. Eighteen dropped out after giving informed consent, while 38 discontinued later in the survey. One outlier on the age variable (who chose the age category 80 years or older) was removed from all analyses. This resulted in a total sample size of 136. The sample size for specific analyses varied somewhat due to slight variations in missing data for the different variables.

The demographic characteristics of the sample are found in Table 1. The age ranged from 18 to 65 years (*n* = 136, *M* = 31.90, *SD* = 9.48). The respondents were, on average, approximately 25 years old when they started to provide sexual services (*n* = 104, *M* = 24.71, *SD* = 8.45) and had provided sexual services for approximately 6 years (*n* = 104, *M* = 6.20, *SD* = 6.52). Most respondents were cis women, had Finnish citizenship, and worked only from and/or in Finland. Furthermore, most sex workers (82.0%) had at least graduated from high school (10–12 years of education). Being in a relationship (i.e., in a relationship, cohabiting, marriage, or other) was slightly more common (55.9%) than being single (44.1%). Most (71.2%) reported their financial situation as good or very good. The median interval for monthly gross income from sex work only was 1,500–1,999€, while the median interval for total monthly gross income was 2,000–2,499€.

**Table 1:** Demographic characteristics of the sex worker sample

Variable	*n* (%)
Gender	136
Man	11 (8.1)
Woman	108 (79.4)
Transman	0 (0.0)
Transwoman	3 (2.2)
Non‐binary	13 (9.6)
Other	1 (0.7)
Sexual orientation	136
Heterosexual	64 (47.1)
Lesbian/gay	2 (1.5)
Bisexual	33 (24.3)
Pansexual	30 (22.1)
Asexual	6 (4.4)
Other	1 (0.7)
Relationship status	136
Single	60 (44.1)
In a relationship	33 (24.3)
Cohabiting	16 (11.8)
Married	16 (11.8)
Other	11 (8.1)
Has children, yes	57 (41.9)
Birth country	136
Finland	127 (93.4)
Other	9 (6.6)
Work country	136
Only from/in Finland^a^	126 (92.6)
Also somewhere else	10 (7.4)
Residence in Finland	134
Finnish citizen	124 (92.5)
Permanent residence permit	3 (2.2)
Temporary residence permit	3 (2.2)
No residence permit	0 (0.0)
Did not want to say	4 (2.9)
Highest educational level	134
No education	0 (0.0)
Primary (6 or fewer years)	4 (3.0)
Secondary (7–9 years)	20 (14.9)
High school/vocational school (10–12 years)	55 (41.0)
University/applied university (13 years or more)	55 (41.0)
Work besides sex work^b^	136
Other paid full‐time/part‐time work	47 (29.0)
Volunteer work	16 (9.9)
Studying/completing an internship	32 (19.8)
Caregiver to parents/children/other family member	6 (3.7)
Something else	23 (14.2)
No other work	38 (23.5)
Monthly gross income – sex work	104
0–1,499€	49 (47.1)
1,500–2,999€	22 (21.2)
3,000–4,499€	20 (19.2)
4,500€ or more	13 (12.5)
Monthly gross income – total	104
0–1,499€	37 (35.6)
1,500–2,999€	22 (21.1)
3,000–4,499€	16 (15.4)
4,500€ or more	29 (27.9)
Financial situation	104
Bad, had to take a loan	5 (4.8)
Tight, not enough money	3 (2.9)
Quite tight, just enough money	22 (21.2)
Good, but spends all money	22 (21.2)
Very good, saves money	52 (50.0)

*Note*: *N =* 136. The number of respondents (*n*) varied in the different variables due to dropout.

^a^ Includes media‐based services provided from Finland and/or in‐person services provided in Finland.

^b^ Respondents could choose several response options. The percentages reflect the frequency distribution between the different response options.

### Measures

This study was part of a more extensive collection of data on sex workers' health. Responses were given on a Likert‐type scale or as open‐ended responses, and all items were mandatory. A detailed overview of study items can be found as supplementary material.

#### Demographic variables and sex work variables

We asked respondents to report their age, gender, sexual orientation, relationship status, country of birth, residence in Finland, educational level, and whether they had children. Furthermore, we inquired about the respondents' sex work (e.g., what sexual services they were providing and where, whether the sex worker often thinks about quitting providing sexual services, and the reasons behind it), working situation (e.g., work besides providing sexual services), salary, and financial situation.

#### Professional agency

We used a slightly modified version of the Pearlin Mastery Scale (PMS) to measure the sex workers' professional agency. The original scale, with seven items, is a widely used measure of how much an individual perceives their life to be under their control or controlled by external factors (Pearlin & Schooler, 1978). However, we modified the scale to suit our research questions. We added three self‐made items probing the ability to decide what to do at work, when to stop providing sexual services, and whether things work out the way they want them to in their work. Separately from the modified scale, we asked how much the respondents agreed with two agency statements concerning voluntariness in their decisions to start and continue providing sexual services. These items were added to measure the voluntariness of sex work and to investigate the correlation between the two items and the Pearlin Mastery Scale. All agency items were rated on a four‐point Likert scale, ranging from 1 (*strongly disagree*) to 4 (*strongly agree*). We reversed negatively phrased items and summed the items together into a score ranging from 10 to 40, where a higher score indicated higher professional agency. The internal reliability of the scale was excellent (Cronbach's α = 0.94).

#### Quality of life

To measure the self‐perceived quality of life during the last 2 weeks, we used the short form of the World Health Organization Quality of Life Instrument (WHOQOL‐BREF; Whoqol Group, 1998). The WHOQOL‐BREF contains 26 questions; four dimensions (physical health, mental health, a social dimension, and an environmental dimension) are measured through 24 questions. The social domain includes, for instance, a question about support from friends, while the environmental domain includes, for instance, questions about health services and transport. The two remaining questions measure general health and overall quality of life. Every item is measured on a five‐point Likert‐type scale ranging from 1 (*very poor/very dissatisfied/not at all/never*) to 5 (*very good/very satisfied/an extreme amount/extremely/completely/always*). We reversed negatively phrased items and summed the items together to form a sum score ranging from 26 to 130, where a higher score indicated a higher quality of life. To compare results across studies, we further standardized the domain scores using the formula provided in the scoring manual, resulting in domain scores with a possible range of 0–100. The WHOQOL‐BREF has been demonstrated to be a reliable and valid brief method for assessing quality of life (Whoqol Group, 1998) and has previously been used to study female sex workers' quality of life (Khodabakhshi Koolaee & Damirchi, 2016; Picos, González & de la Iglesia Gutiérrez, 2018; Pinedo González, Palacios Picos & de La Iglesia Gutiérrez, 2021). In the current study, the internal reliability of the scale was excellent (Cronbach's α = 0.96).

#### Problematic alcohol use

We used a slightly modified version of the Alcohol Use Disorders Identification Test (AUDIT) to assess the sex workers' problematic alcohol use during the last 6 months. The original scale was developed by Saunders, Aasland, Babor, de la Fuente, and Grant (1993). We removed questions concerning frequency and amount of alcohol use to focus only on problematic alcohol use and minimize the stigma the sex workers would perceive while answering the questions. Because the requirement to participate in our study was that the sex worker had provided sexual services for the past 6 months, we also limited the time to the last 6 months. Our modified version began with a self‐made question regarding whether the sex worker had used alcohol during the last 6 months, with the response options *Yes* and *No*. Seven questions (items 4–10 from the original scale) were then asked only if the response was *Yes*. Of these, two response options (*No* and *Yes, but not in the past 6 months*) were scored as 0, and one (*Yes, during the past 6 months*) was scored as 4. The five remaining questions were scored on a Likert scale ranging from 0 (*never*) to 4 (*daily or almost daily*). The items were summed to a variable ranging from 0 to 28, where a higher score indicated more problematic alcohol use. In the current study, the internal reliability of the scale was good (Cronbach's α = 0.88).

#### Problematic drug use

We used a slightly modified version of the Drug Use Disorder Identification Test (DUDIT) to assess the sex workers' problematic drug use during the last 6 months. The original scale was developed by Berman, Bergman, Palmstierna, and Schlyter (2005). As with the AUDIT, we limited the assessed time to 6 months and modified it to focus on problematic drug use. Our modified version began with a self‐made question regarding whether the sex worker had used drugs during the last 6 months, with the response options *Yes* and *No*. We did not specifically define types of drugs, but the instructions provided to respondents stated that medications were not considered drugs if they were prescribed by a doctor and taken in the prescribed doses. Seven questions (items 4–10 from the original scale) were then asked only if the response was *Yes*. Of these, two response options (*No* and *Yes, but not in the past 6 months*) were scored as 0, and one (*Yes, during the past 6 months*) was scored as 4. The five remaining questions were scored on a Likert scale ranging from 0 to 4. The points were summed to a variable ranging from 0 to 28, where a higher score indicated a higher level of problematic drug use. In the current study, the internal reliability of the scale was good (Cronbach's α = 0.85).

### Procedure

Before the data collection, we piloted the survey with four sex workers contacted via Pro‐tukipiste (an organization supporting sex workers) to ensure that the items would not be stigmatizing for the sex workers. These responses were not included in the final sample. The feedback comments concerned, for instance, the order of response options and suggestions for rewordings. We modified the survey following the feedback before we collected data.

Our study lasted from February 10, 2022, to March 6, 2022. We recruited respondents through Pro‐tukipiste and FTS Finland (a network for sex workers in Finland), via social media platforms (Facebook and Instagram), and other Internet forums for sex workers (e.g., Seksisaitti). We also used snowball sampling by contacting sex workers to invite them to participate and share the link to our survey with other sex workers. We contacted both publicly known sex workers and sex workers found through different Instagram accounts (e.g., suomionlyfans) and hashtags (e.g., #ofsuomi). On Instagram, we contacted only individuals with information about their sexual services in their Instagram bios.

By following the link, interested respondents were redirected to our online survey on a secure online platform. Respondents could choose between filling out the survey in Finnish or English. The respondents gave informed consent before participating. Furthermore, we informed them about their complete anonymity and the possibility of discontinuing the survey at any point without giving a reason for doing so. As an incentive to participate in the study, respondents were offered the opportunity to participate in a lottery of three gift cards, each worth 50€, redeemable at one of two online stores in Finland, upon completing the survey. Participants could enter the lottery by providing their email addresses, which were collected and stored separately from their survey responses to ensure confidentiality. These email addresses were deleted immediately after the lottery concluded. Participation in the lottery was voluntary, and participants were fully informed about how their data would be managed.

### Statistical analyses

All statistical analyses were performed using IBM SPSS Statistics 28. First, we checked the data for outliers and deleted one extreme outlier (with a score of 4.19 standard deviations from the mean) found in the AUDIT analysis. Second, we conducted bootstrapped correlational analyses for all study variables. We used point‐biserial correlations for dichotomized variables. After that, we checked whether the dependent variables met the assumptions for linear regressions. Since all assumptions were not met, we performed seven robust analyses with bootstrapped confidence intervals, one per dependent variable, with the sum score of the Pearlin Mastery Scale as an independent variable in each of the analyses. The seven dependent variables were the four WHOQOL‐BREF subscales and the sum scores for the WHOQOL‐BREF, AUDIT, and DUDIT.

## RESULTS

### Descriptive results

Table 2 displays frequencies and percentages of responses for the sex work variables. Providing in‐person services (89.4%) was more common than media‐based services (48.1%). Of the sex workers, 62.5% offered services online or in person, while 37.5% offered services both online and in person.

**Table 2:** Descriptive information of sex workers' client interactions, services, work location, and reasons to quit

Variable	*n* (%)
Typical client gender^a^	104
Man	99 (95.2)
Woman	3 (2.9)
Other	2 (1.9)
Interaction with client^b^	104
No interaction (e.g., photos on a platform)	20 (9.4)
Interaction, online/by phone	51 (23.9)
Interaction in person	95 (66.7)
No physical or sexual contact (e.g., stripping on a scene)	13 (6.1)
Physical contact, no sexual contact	44 (20.7)
Sexual contact	85 (39.9)
Media‐based services, yes^c^	50 (48.1)
Type of media‐based services^b^	
Photos/videos	40 (42.6)
Webcamming	19 (20.2)
Phone calls/messages	31 (33.0)
Other	4 (4.3)
In‐person services, yes^c^	93 (89.4)
Type of in‐person services^b^	
Full services^d^	70 (25.2)
Escorting	43 (15.5)
Massage	33 (11.9)
Dance/stripping	18 (6.5)
Girl/boyfriend experience	59 (21.2)
Sugar dating	17 (6.1)
Fetish sessions/BDSM	35 (12.6)
Other	3 (1.1)
Service location^b^	104
Only services online/by phone	11 (4.3)
Own home	49 (19.0)
Client's home	53 (20.5)
Brothel	1 (0.4)
Strip club/erotic bar	7 (2.7)
Massage parlor	1 (0.4)
Hotel	54 (20.9)
Studio (e.g., sadomasochism studio)	14 (5.4)
Street	4 (1.6)
Car	26 (10.1)
Rented apartment	31 (12.0)
Somewhere else	7 (2.7)
Often thinking about quitting	104
Not at all true	64 (61.5)
Slightly true	25 (24.0)
Very true	15 (14.4)
Reasons to quit^b^, ^e^	40
Try a new job	5 (4.3)
Better job offer	0 (0.0)
This is only a temporary job	16 (13.9)
Not paid enough	7 (6.1)
Not liking my job	20 (17.4)
Wanting to study	8 (7.0)
Expecting a child	0 (0)
Affecting relationship/s	13 (11.3)
Too much stigma	10 (8.7)
Physical/mental illness	23 (20.0)
Retiring soon	4 (3.5)
Other reason	9 (7.8)

*Note*: *N* = 136. Of these, 104 (who did not drop out) provided descriptive information about their work situation.

^a^ Reflects the most common client.

^b^ Respondents could choose several response options. The percentages reflect the frequency distribution between the different response options.

^c^ Reflects the number of respondents providing this type of service.

^d^ Full service indicates a service that includes intercourse.

^e^ Includes only respondents who answered slightly true or very true to the variable “often thinking about quitting.”

Descriptive statistics for scales and variables are found in Table 3. The majority, 85.6%, responded “somewhat agree” or “strongly agree” to the statement that the decision to start providing sexual services was completely voluntary, and 87.4% responded “somewhat agree” or “strongly agree” to the statement that the decision to continue is completely voluntary. Most sex workers rated their quality of life and general health as good or very good, 70.8% and 73.2%, respectively. In the past 6 months, 68.3% of the sex workers had used alcohol, while 23.8% had used drugs at some moment.

**Table 3:** Descriptive statistics for scales and variables measuring professional agency, quality of life, and problematic substance use

Scale	*n*	*M*	*SD*	Range	Cronbach's α
Decision to start	111	3.51	0.87	1–4	–
Decision to continue	111	3.61	0.73	1–4	–
Pearlin Mastery Scale	111	32.64	7.63	10–40	0.94
WHOQOL‐BREF	120	100.10	21.68	41–130	0.96
Psychological	120	66.38	23.27	0–100	0.90
Physical	120	72.66	23.89	0–100	0.91
Social	120	69.90	26.24	0–100	0.84
Environmental	120	76.27	20.37	0–100	0.87
Overall quality of life	127	3.86	1.03	1–5	–
General health	127	3.77	1.12	1–5	–
AUDIT	68	3.03	4.46	0–28	0.88
DUDIT	24	7.25	7.63	0–28	0.85

*Note*: *N =* 136. The number of respondents (*n*) varied in the different variables due to dropout. A higher score indicated a higher level of voluntariness, professional agency, quality of life, and problematic substance use. Decision to start/continue = Likert item assessing to what extent respondents perceived that their decision to start/continue providing sexual services was completely voluntary. The Pearlin Mastery Scale was used to measure professional agency. WHOQOL‐BREF = The World Health Organization Quality of Life–BREF; AUDIT = modified version of the Alcohol Use Disorders Identification Test (including only those who reported alcohol use during the past 6 months); DUDIT = modified version of the Drug Use Disorders Identification Test (including only those who reported drug use during the past 6 months).

### Correlation analyses

Table 4 contains the results from the initial correlation analyses.

**Table 4:** Correlations and bootstrapped confidence intervals for scales and variables

Variable	WHOQOL‐BREF	95% CI	AUDIT	95% CI	DUDIT	95% CI	PMS	95% CI
WHOQOL‐BREF	1							
AUDIT	−0.37**	[−0.60, −0.09]	1					
DUDIT	−0.63**	[−0.83, −0.36]	0.52*	[0.10, 0.93]	1			
PMS	0.86***	[0.78, 0.91]	−0.38**	[−0.62, −0.05]	−0.69***	[−0.87, −0.46]	1	
Decision to start	0.78***	[0.69, 0.84]	−0.41**	[−0.66, −0.07]	−0.57**	[−0.83, −0.22]	0.86***	[0.79, 0.90]
Decision to continue	0.76***	[0.66, 0.84]	−0.30*	[−0.56, −0.00]	−0.54**	[−0.84, −0.20]	0.86***	[0.79, 0.91]
Age	0.15	[−0.02, 0.30]	−0.05	[−0.27, 0.22]	−0.18	[−0.58, 0.17]	0.24*	[0.08, 0.39]
Single^a^	−0.21*	[−0.36, −0.03]	0.06	[−0.18, 0.31]	−0.08	[−0.48, 0.35]	−0.18	[−0.36, −0.01]
Heterosexual^b^	0.04	[−0.14, 0.23]	0.22	[−0.04, 0.44]	0.44*	[0.07, 0.79]	−0.03	[−0.23, 0.15]
Children^c^	−0.02	[−0.21, 0.16]	0.09	[−0.16, 0.35]	0.04	[−0.30, 0.43]^i^	−0.09	[−0.28, 0.10]
Birth country^d^	0.27**	[0.10, 0.43]	0.01	[−0.17, 0.14]^g^	−0.07	[−0.41, 0.23]^j^	0.26**	[0.03, 0.46]
Work country^e^	0.14	[−0.08, 0.34]	−0.06	[−0.32, 0.13]^h^	0.13	[−0.14, 0.37]^k^	0.16	[−0.02, 0.35]
Education	0.51***	[0.35, 0.65]	−0.46***	[−0.63, −0.20]	−0.46*	[−0.79, −0.06]	0.58***	[0.39, 0.72]
Years providing	−0.36***	[−0.54, −0.15]	0.38**	[0.15, 0.56]	0.36	[−0.03, 0.68]	−0.47***	[−0.65, −0.28]
Age start providing	0.40***	[0.21, 0.56]	−0.31*	[−0.50, −0.08]	−0.37	[−0.76, −0.12]	−0.56***	[0.44, 0.67]
Does work besides	0.25**	[0.05, 0.44]	−0.13	[−0.39, 0.13]	−0.19	[−0.61, 0.21]	0.27**	[0.06, 0.47]
Does online sex work	0.15	[−0.04, 0.33]	−0.03	[−0.25, 0.21]	0.21	[−0.21, 0.60]	0.15	[−0.04, 0.33]
Does in‐person sex work	−0.06	[−0.22, 0.12]	0.13	[−0.01, 0.25]	−0.15	[−0.63, 0.35]^l^	−0.15	[−0.28, 0.01]
Thinking about quitting work^f^	−0.61***	[−0.73, −0.47]	0.19	[−0.06, 0.43]	0.16	[−0.24, 0.55]	−0.56***	[−0.69, −0.40]
Monthly gross income – sex work	0.39***	[0.28, 0.47]	−0.17	[−0.35, 0.02]	−0.17	[−0.52, 0.14]	0.28**	[0.17, 0.40]
Monthly gross income – total	0.46***	[0.34, 0.58]	−0.21	[−0.40, −0.01]	−0.30	[−0.71, 0.02]	0.37***	[0.24, 0.49]
Financial situation	0.58***	[0.44, 0.70]	−0.21	[−0.46, 0.05]	−0.50*	[−0.74, −0.17]	0.44***	[0.28, 0.58]

*Note*: Unless otherwise mentioned, the exact number of bootstrapped samples for the confidence intervals was 2,000. WHOQOL‐BREF = sum score based on all 26 of the World Health Organization Quality of Life–BREF items; AUDIT = sum score based on a modified version of the Alcohol Use Disorders Identification Test (including only respondents who had used alcohol during the past 6 months); DUDIT = sum score based on a modified version of the Drug Use Disorders Identification Test (including only respondents who had used drugs during the past 6 months); PMS = sum score variable based on a modified version of the Pearlin Mastery Scale. Decision to start/continue = Likert item assessing to what extent respondents perceived that their decision to start/continue providing sexual services was completely voluntary.

^a^ 1 = single, 0 = relationship.

^b^ 1 = heterosexual, 0 = other.

^c^ 1 = has children, 0 = has no children.

^d^ 1 = Finland, 0 = other.

^e^ 1 = only Finland, 0 = other countries as well as Finland.

^f^ 1 = responses *very true* or *slightly true* and 0 = responses *not at all true* on the item “I often think about quitting my work.”

^g^ Based on 1,997 samples.

^h^ Based on 1,989 samples.

^i^ Based on 1,991 samples.

^j^ Based on 1,919 samples.

^k^ Based on 1,900 samples.

^l^ Based on 1,759 samples.

* *p* < 0.05;

** *p* < 0.01;

*** *p* < 0.001.

The Pearlin Mastery Scale was positively correlated with the WHOQOL‐BREF (*r* = 0.86) and negatively associated with the AUDIT (*r* = −0.38) and DUDIT (*r* = −0.69), indicating that more professional agency was associated with a higher quality of life and lower levels of problematic substance use. These scales were consequently used in the regression analyses presented below.

The WHOQOL‐BREF further correlated positively with the two statements regarding the respondent's feeling that the decision to start (*r* = 0.78) and to continue (*r* = 0.76) providing sexual services was completely voluntary, indicating that a higher level of voluntariness was associated with a higher quality of life. These two statements were also negatively correlated with the AUDIT (*r* = −0.41 and −0.30) and DUDIT (*r* = −0.57 and −0.54), indicating that a higher level of voluntariness was also associated with a lower level of problematic substance use.

The Pearlin Mastery Scale and WHOQOL‐BREF were further associated with several demographic variables, for instance, education (*r* = 0.58 and 0.51), sex work income (*r* = 0.28 and 0.39), total income (*r* = 0.37 and 0.46), and birth country (*r* = 0.26 and 0.27). This indicates that higher levels of professional agency and quality of life were associated with higher educational levels, higher income, and being born in Finland. The AUDIT was negatively associated with education (*r* = −0.46), indicating that more problematic alcohol use was associated with a lower level of education. The DUDIT was negatively associated with both education (*r* = −0.46) and financial situation (*r* = −0.50), indicating that more problematic drug use was associated with lower education and poorer financial situation.

### Regression analyses

Our results from the robust regression analyses for the WHOQOL‐BREF, AUDIT, and DUDIT are shown in Table 5. All models were significant. Professional agency explained 73.2% of the variance in quality of life (47.6–70.1% for the specific subscales), while it explained 14.2% of the variance in problematic alcohol use and 47.3% of the variance in problematic drug use.

**Table 5:** Regression results for professional agency, quality of life, and problematic substance use

Dependent variable	*R* ^2^	Adj. *R* ^2^	*F*	*B*	Robust SE	β	*t*	*p*	95% CI
WHOQOL‐BREF	0.73	0.73	297.72	2.48	0.12	0.86	17.26	<0.001	[2.24, 2.69]
Psychological	0.60	0.59	160.85	2.39	0.18	0.77	12.68	<0.001	[2.02, 2.75]
Physical	0.56	0.55	137.68	2.40	0.21	0.75	11.73	<0.001	[1.98, 2.78]
Social	0.48	0.47	98.83	2.34	0.28	0.69	9.94	<0.001	[1.81, 2.89]
Environmental	0.70	0.70	255.81	2.27	0.12	0.84	16.00	<0.001	[2.05, 2.48]
AUDIT	0.14	0.13	10.89	−0.26	0.10	−0.38	−3.30	0.002	[−0.44, −0.03]
DUDIT	0.47	0.45	19.74	−0.62	0.10	−0.69	−4.44	<0.001	[−0.85, −0.38]

*Note*: A modified version of the Pearlin Mastery Scale was used to measure professional agency, and its sum score represented the independent variable in all analyses. Note that β corresponds to the correlation coefficient in the correlational analyses. WHOQOL‐BREF = sum score of the World Health Organization Quality of Life–BREF; AUDIT = sum score of a modified version of the Alcohol Use Disorders Identification Test; DUDIT = sum score of a modified version of the Drug Use Disorders Identification Test. The confidence intervals were based on bias‐corrected and accelerated bootstrap samples (*n* = 2,000).

## DISCUSSION

In the present study, we investigated whether sex workers' professional agency was associated with their quality of life and problematic substance use by surveying sex workers who provide sexual services in Finland. We also explored the associations between different demographic aspects (i.e., financial situation and education level), professional agency, quality of life, and problematic substance use. To our best knowledge, the present study was the first quantitative study in Finland to examine how sex workers' professional agency is related to the quality of life and problematic substance use and the first to include media‐based sex workers.

### Sample characteristics

By using a broad definition of sex work and being inclusive of all genders, we aimed to recruit a demographically diverse sample. However, in line with previous research from Finland and abroad (Birch, 2015; Liitsola, Kauppinen, Nikula, *et al*., 2013; TAMPEP, 2010), most respondents in the current study identified as cis women.

The distribution of country of birth in our study differed from previous estimates. In our sample, 93% were born in Finland. For instance, the European Network for the Promotion of Rights and Health Among Migrant Sex Workers estimated that roughly two‐thirds of sex workers in Finland are migrants (TAMPEP, 2010). In a previous study by Liitsola, Kauppinen, Nikula, *et al*. (2013), the number of Finnish‐born sex workers was 28%. Possible explanations for our result could be that foreign sex workers engage less in the Finnish sex worker networks we used for recruitment or that there were language barriers, as our study was available only in English and Finnish. Liitsola, Kauppinen, Nikula, *et al*. (2013) also experienced challenges recruiting foreign sex workers and noticed distrust toward confidentiality and the research purpose among some foreign sex workers. There seems to be a general problem in reaching foreign‐born sex workers, who might be more structurally vulnerable and marginalized. In our sample, being born outside Finland was associated with lower quality of life and less professional agency. Though research on this topic is scarce, it is also likely that the COVID‐19 pandemic affected the market for foreign‐born sex workers in Finland, as international traveling became increasingly difficult.

Around half of the respondents (48.1%) provided media‐based services, while only one‐tenth provided media‐based services only. A reason for the low number of media‐based services only could be that some of these individuals do not consider themselves sex workers and, therefore, chose not to participate in our study. For instance, the label *adult content creator* has sometimes been used instead of sex worker when referring to individuals providing services through various online platforms (Milan, 2022; Uttarapong, Bonifacio, Jereza & Wohn, 2022).

Concerning their financial situation, almost three‐fourths of the respondents rated their financial situation as good or very good. This is higher than in Liitsola, Kauppinen, Nikula, *et al*. (2013), where fewer than two‐thirds of the respondents reported a good financial situation. The median interval for monthly gross income from only sex work was 1,500–1,900€, while the median interval for the total monthly gross income was 2,000–2,499€. This is somewhat lower than the median salary for full‐time work in Finland (monthly gross income of 3,228€ in 2020; Official Statistics of Finland, 2022). A better financial situation and a higher income (both sex work only and total) were associated with a higher level of professional agency and a higher quality of life. This finding is in line with a study on the general Finnish population, in which the risk of poor quality of life decreased as income increased (Vaarama, Moisio & Karvonen, 2010). Similar findings have also been reported among sex workers in other countries. For instance, a recent study on sex workers in Greece reported that a poor financial situation was associated with a decline in both physical and mental health (Drydakis, 2021).

Concerning educational level, around two‐fifths (41.0%) of the respondents in our study had a university education, while an additional two‐fifths (41.0%) had a high school or vocational school education. This is somewhat lower in comparison with women aged 30–34 in the general population (i.e., the demographic group closest to our sample), of whom around half (47.6%) are estimated to have a university degree (Tilastokeskus, 2022). Note, however, that our sample was more heterogeneous. Higher education was significantly associated with higher quality of life and more professional agency. The findings concerning quality of life correspond to research on the Finnish population, where researchers reported that the main risk factors for low quality of life included low educational level and unemployment (Vaarama, Moisio & Karvonen, 2010).

An interesting finding regarding sample characteristics was that a small proportion (4.4%) of our sample of sex workers considered themselves asexual. This indicates that an absence of (or very low) sexual attraction toward others or interest in having sex is not an obstacle to working as a sex worker. Interestingly, this number is also noticeably larger than recent population‐based estimates of asexuality (0.7%; Källström, Nousiainen, Jern, Nickull & Gunst, 2022), though it could be that our estimate is not reliable due to the much smaller sample size.

### Quality of life

Most of the sex workers in our sample rated their quality of life as either good or very good. Scores on the two specific items measuring the respondents' perception of their general quality of life and general health were comparable to the general Finnish population (Vaarama, Moisio & Karvonen, 2010). However, the respondents in our study scored lower in each subdomain of quality of life. Moreover, when comparing our results with women aged 25–44 in the general Finnish population (as the mean age in our sample was 32 years), the difference in perceived quality of life was even greater. However, it is important to note that the standard deviations for each domain were larger in our sample, which means that there was more variance. It is also worth noting that almost all respondents in our sample were born in Finland, which means that our results likely do not generalize to the foreign‐born sex workers, who have been estimated to make up most of the sex workers in Finland (TAMPEP, 2010).

To our knowledge, there are only two studies on sex workers conducted abroad in which the WHOQOL‐BREF has been used as the measure of quality of life and in which the authors have reported the mean scores: one study conducted in Iran (Khodabakhshi Koolaee & Damirchi, 2016) and one in India (Shukla & Mehrotra, 2014). Compared with these two studies, the mean scores for sex workers in Finland were higher on all four dimensions of quality of life. One explanation for a higher quality of life for sex workers in Finland could be that the countries' cultures and laws regarding sex work differ. For instance, (native) sex workers in Finland do not have to fear being arrested for providing sexual services. This might also make reporting violent clients to authorities easier, potentially increasing the quality of life. Another explanation for this result is that the general standard of living is considerably higher in Finland compared with Iran and India (WorldData.info, 2022).

### Problematic substance use

Around one‐fourth of the respondents in our study had used some drug during the past 6 months. This estimate is higher than that for the general Finnish population, of whom 8% reported using drugs during the past year (assessed in 2018; Karjalainen, Hakkarainen & Salasuo, 2019). This was also the case when comparing our results with the age‐group 25–34 years, in which drug use during the past year was reported by 18%. It is also important to note that we measured the sex workers' drug and alcohol use in the last 6 months, while the study on the general Finnish population measured the use in a year, meaning that the difference could be even larger.

Regarding alcohol use, sex workers' alcohol use seems comparable to that of the general Finnish population. In our sample, roughly two‐thirds had used alcohol in the past 6 months, while 85% of Finns aged 15–79 had used alcohol at some point in 2016 (Mäkelä, Härkönen, Lintonen & Tigerstedt, 2018). As with drug use, it should be noted that we assessed alcohol use only during the past 6 months, indicating that the proportion of sex workers who use alcohol would probably be larger if it had been assessed for a year.

### Professional agency, quality of life, and problematic substance use

In line with both of our hypotheses, a higher level of professional agency among the sex workers was associated with a higher quality of life and with lower levels of problematic substance use. In the regression analysis, professional agency explained as much as 73% of the variance in quality of life, indicating that professional agency is very central to sex workers' quality of life. For the specific subscales for quality of life, professional agency was the most central to the environmental subscale (inquiring, e.g., about health services) and the least central to the social subscale (inquiring, e.g., about support from friends). It seems as though professional agency is especially important when it comes to environmental aspects of quality of life, such as physical safety, health and social care, and financial resources. Further, both items measuring the voluntariness of the decision to start providing and continue providing sexual services significantly and strongly correlated with quality of life. This indicates that a higher level of perceived voluntariness in the decision to start or continue providing sexual services is associated with a higher quality of life. The Pearlin Mastery Scale correlated strongly with both items, indicating that professional agency and perceived voluntariness in sex work are closely related concepts.

The association between professional agency and quality of life is consistent with previous research from the United States, which has found positive associations between sex workers' agency and related concepts (e.g., physical health and social support; Buttram, Surratt & Kurtz, 2014; Dalla, Xia & Kennedy, 2003). It is worth noting that the aforementioned studies focused on street‐based sex workers, who are among the most marginalized subgroups of sex workers. Participants in our study are likely to face fewer structural barriers to, for example, occupational health and safety. Thus, our findings suggest that professional agency is relevant in less marginalized samples of sex workers.

### Clinical implications

Our results underscore the importance of professional agency to the well‐being of sex workers, suggesting that public health and social service agencies should prioritize policies and interventions that enhance professional agency. Efforts to protect sex workers from exploitation and to create a supportive legal and social environment could play an important role in empowering sex workers to make informed decisions about their work and lives, potentially improving their overall quality of life. Additionally, support services that provide access to necessary resources can further enhance professional agency among sex workers.

## LIMITATIONS AND FUTURE DIRECTIONS

The present study includes some limitations to be considered when interpreting the results. One concern is that almost all respondents were Finnish cis women, meaning that conclusions about other genders and foreign‐born sex workers in Finland must be drawn with caution. Future studies on professional agency and sex work should prioritize reaching these populations. Including a more diverse population could provide a broader understanding of the role of professional agency across different groups within the sex work community.

Another concern is that collecting data on sex work may be particularly delicate due to the stigma associated with sex work, as this could lead to biases in who chooses to participate. It could be that sex workers with higher well‐being are more interested in participating in the study, giving a more positive image of sex work. On the other hand, internalized stigma might also affect the responses so that sex workers respond following their beliefs of people's opinions about sex work, thus reporting lower quality of life. Moreover, as an incentive to participate in the study, respondents were given the opportunity to participate in a lottery for online gift cards worth 50€. While participation in the lottery required providing an email address (stored separately from the survey responses), respondents who preferred not to provide any personal information could choose not to participate in the lottery and still complete the survey. Nevertheless, we acknowledge that offering an incentive may have slightly affected the respondent pool by potentially attracting participants who are particularly motivated by such rewards.

Among methodological limitations, the current study was cross‐sectional, allowing no conclusions regarding causality. Future longitudinal studies could examine how professional agency develops over time and its long‐term effects on sex workers' well‐being and quality of life. It should also be noted that to suit our sample better, we used slightly modified versions of the original validated scales. However, the internal reliability of the modified scales was good.

Finally, future research on professional agency in sex work could explore how changes in legislation and policy, particularly those aimed at improving sex workers' rights and access to services, affect professional agency and quality of life.

## CONCLUSION

We found that sex workers' professional agency was strongly and positively associated with quality of life and negatively associated with problematic substance use. Sex workers in Finland appear to have a relatively high quality of life and use amounts of alcohol that compare to the general Finnish population. Drug use was, however, more common among sex workers than the general Finnish population. The results of the present study may be important in ensuring support of sex workers' agency to achieve a high quality of life and decrease the risk of problematic substance use. However, as the present study mainly included Finnish‐native female sex workers, conclusions about foreign‐born sex workers and sex workers of other genders must be drawn with caution.

## Supporting information


**Data S1.** Survey items.

## Data Availability

The data that support the findings of this study are available upon reasonable request from the corresponding author. The data are not publicly available due to privacy or ethical restrictions.
