# Supplementary material for: Sex workers' professional agency, quality of life, and problematic substance use in Finland
Source: Scand J Psychol. 2024 Sep 15;66(1):131–40. doi: 10.1111/sjop.13070 (PMC11735243; doi:10.1111/sjop.13070)
Supplement: Supplementary file 1 — Data S1. Survey items. [file SJOP-66-131-s001.pdf]

Survey Items

Items and Response Options in the Current Survey

Item block	Question	Response Options
Demographics	Your current age in years:	18; 19; 20; 21; 22; 23; 24; 25; 26; 27; 28; 29; 30; 31; 32; 33; 34; 35; 36; 37; 38; 39; 40; 41; 42; 43; 44; 45; 46; 47; 48; 49; 50; 51; 52; 53; 54; 55; 56; 57; 58; 59; 60; 61; 62; 63; 64; 65; 66; 67; 68; 69; 70; 71; 72; 73; 74; 75; 76; 77; 78; 79; 80 years or older
	Your gender:	Man; Woman; Transman; Transwoman; Non-binary; Other, what?
	Your sexual orientation:	Heterosexual; Homosexual; Bisexual; Pansexual; Asexual; Other, what?
	Your relationship status:	Single; In a relationship; Cohabiting; Married; Other, what?
	Do you have children?	Yes; No
	Your country of birth:	Open-ended question
	Do you currently provide sexual services in some other country/countries than Finland?	No, only in Finland (in person) or from Finland (online); Yes, also somewhere else than Finland
	Please write which other country/countries than Finland you are currently providing sexual services in:	Open-ended question
	Choose the option that suits you best:	I am a Finnish citizen; I have a permanent residence permit in Finland; I have a temporary residence permit in Finland; I do not have a residence permit in Finland; I do not want to say
	What is the highest level of education you have completed?	No education; Primary (6 years or less); Secondary (7-9 years); High school or vocational school (10-12 years); University or applied university (13 years or more)
	Besides providing sexual services, choose which of the following work-related options suits you: (You can choose one or several answers that suits you best).	I have other paid full-time or part-time work; I do volunteer work; I am studying or completing an internship; I am a caregiver (to parents, children or other family member); Something else; I do not have any other work
WHOQOL-BREF	How would you rate your quality of life?	Very good; Good; Neither poor nor good; Poor; Very poor
	How satisfied are you with your health?	Very satisfied; Fairly satisfied; Neither satisfied nor dissatisfied; Fairly dissatisfied; Very dissatisfied
	To what extent do you feel that physical pain prevents you from doing what you need to do?	An extreme amount; A great deal; A moderate amount; A small amount; Not at all
	How much do you need any medical treatment to function in your daily life?	An extreme amount; A great deal; A moderate amount; A small amount; Not at all

How much do you enjoy life?	An extreme amount; A great deal; A moderate amount; A small amount; Not at all
To what extent do you feel your life to be meaningful?	An extreme amount; A great deal; A moderate amount; A small amount; Not at all
How well are you able to concentrate?	Extremely; Very; Moderately; Slightly; Not at all
How safe do you feel in your daily life?	Extremely; Very; Moderately; Slightly; Not at all
How healthy is your physical environment?	Extremely; Very; Moderately; Slightly; Not at all
Do you have enough energy for everyday life?	Completely; To a great extent; Somewhat; Slightly; Not at all
Are you able to accept your bodily appearance?	Completely; To a great extent; Somewhat; Slightly; Not at all
Have you enough money to meet your needs?	Completely; To a great extent; Somewhat; Slightly; Not at all
How available to you is the information you need in your daily life?	Completely; To a great extent; Somewhat; Slightly; Not at all
To what extent do you have the opportunity for leisure activities (hobbies)?	Completely; To a great extent; Somewhat; Slightly; Not at all
How well are you able to get around physically?	Extremely; Very; Moderately; Slightly; Not at all
How satisfied are you with your sleep?	Very satisfied; Fairy satisfied; Neither satisfied nor dissatisfied; Fairly dissatisfied; Very dissatisfied
How satisfied are you with your ability to perform your daily living activities?	Very satisfied; Fairy satisfied; Neither satisfied nor dissatisfied; Fairly dissatisfied; Very dissatisfied
How satisfied are you with your capacity for work?	Very satisfied; Fairy satisfied; Neither satisfied nor dissatisfied; Fairly dissatisfied; Very dissatisfied
How satisfied are you with yourself?	Very satisfied; Fairy satisfied; Neither satisfied nor dissatisfied; Fairly dissatisfied; Very dissatisfied
How satisfied are you with your personal relationships?	Very satisfied; Fairy satisfied; Neither satisfied nor dissatisfied; Fairly dissatisfied; Very dissatisfied
How satisfied are you with your sex life?	Very satisfied; Fairy satisfied; Neither satisfied nor dissatisfied; Fairly dissatisfied; Very dissatisfied
How satisfied are you with the support you get from your friends?	Very satisfied; Fairy satisfied; Neither satisfied nor dissatisfied; Fairly dissatisfied; Very dissatisfied

	How satisfied are you with the conditions of your living place?	Very satisfied; Fairy satisfied; Neither satisfied nor dissatisfied; Fairly dissatisfied; Very dissatisfied
	How satisfied are you with your access to health services?	Very satisfied; Fairy satisfied; Neither satisfied nor dissatisfied; Fairly dissatisfied; Very dissatisfied
	How satisfied are you with your transport?	Very satisfied; Fairy satisfied; Neither satisfied nor dissatisfied; Fairly dissatisfied; Very dissatisfied
	How often do you have negative feelings such as blue mood, despair, anxiety or depression?	Never; Infrequently; Sometimes; Frequently; Always
Voluntariness	I feel like my decision to start providing sexual services was completely voluntary.	Strongly agree; Somewhat agree; Somewhat disagree; Strongly disagree
	I feel like my decision to continue providing sexual services is completely voluntary.	Strongly agree; Somewhat agree; Somewhat disagree; Strongly disagree
Modified Pearlin Mastery Scale	*I can decide what I do in my work.	Strongly agree; Somewhat agree; Somewhat disagree; Strongly disagree
	*I can stop providing sexual services whenever I want to.	Strongly agree; Somewhat agree; Somewhat disagree; Strongly disagree
	*Things never work out the way I want them to in my work.	Strongly agree; Somewhat agree; Somewhat disagree; Strongly disagree
	I cannot solve some of the problems that I have in my work.	Strongly agree; Somewhat agree; Somewhat disagree; Strongly disagree
	Sometimes I feel that I am commanded (pushed around) in my work.	Strongly agree; Somewhat agree; Somewhat disagree; Strongly disagree
	I have little control over the things that happen to me in my work.	Strongly agree; Somewhat agree; Somewhat disagree; Strongly disagree
	Most of the time, I feel helpless in dealing with the problems in my work.	Strongly agree; Somewhat agree; Somewhat disagree; Strongly disagree
	What happens to me in the future, concerning my work, mostly depends on me.	Strongly agree; Somewhat agree; Somewhat disagree; Strongly disagree
	There is little I can do to change most of the important things in my work.	Strongly agree; Somewhat agree; Somewhat disagree; Strongly disagree
	I can do anything in my work when I put my mind to it.	Strongly agree; Somewhat agree; Somewhat disagree; Strongly disagree
Work descriptives	What kind of interaction do you regularly have with your clients? (You can choose one or several answers that suits you best).	No interaction (e.g., photos on a platform); Interaction online or by phone (e.g., chatting, talking or webcamming); Interaction in person but no physical nor sexual contact (e.g., stripping on a scene); Interaction in person with physical but no sexual contact (e.g., massage, kissing or hugging); Interaction in person with sexual contact (e.g., touching genitals or penetration)

What kind of sexual services do you provide online/by phone? (You can choose one or several answers that suits you best).	I do not provide sexual services online/by phone; Photos/videos; Webcamming; Phone calls/messages with client; Other, what?
What kind of sexual services are you providing in person?(You can choose one or several answers that suits you best).	I do not provide sexual services in person, only online/by phone; Full services; Escorting; Massage; Dance/stripping; Girl-/boyfriend experience; Sugar dating; Fetish sessions; Other, what?
Where do you provide sexual services in person?(You can choose one or several answers that suits you best).	I do not provide sexual services in person, only online/by phone; In my home; In client's home; In a brothel; In a strip club/an erotic bar; In a massage parlour; In a hotel; In a studio (e.g., SM); On the street; In a car; In a rented apartment (not my own home); Somewhere else, where?
For how many years have you been providing sexual services?	Less than 1 year; 1; 2; 3; 4; 5; 6; 7; 8; 9; 10; 11; 12; 13; 14; 15; 16; 17; 18; 19; 20; 21; 22; 23; 24; 25; 26; 27; 28; 29; 30; 31; 32; 33; 34; 35; 36; 37; 38; 39; 40; 41; 42; 43; 44; 45; 46; 47; 48; 49; 50; More than 50 years
How old were you when you started providing sexual services?	10 years or younger; 11; 12; 13; 14; 15; 16; 17; 18; 19; 20; 21; 22; 23; 24; 25; 26; 27; 28; 29; 30; 31; 32; 33; 34; 35; 36; 37; 38; 39; 40; 41; 42; 43; 44; 45; 46; 47; 48; 49; 50; 51; 52; 53; 54; 55; 56; 57; 58; 59; 60; 61; 62; 63; 64; 65; 66; 67; 68; 69; 70 years or older
Please, consider how true the following statement is for you: "I often think about quitting my work"	Not at all true; Slightly true; Very true
When you consider stopping providing sexual services, what are the most common reasons to stop? (You can choose one or several answers that suits you best).	I want to try a new work; I got a better work offer; This is only a temporary work; I do not earn enough money; I do not like my work; I want to study; I am expecting a child; It is affecting my relationship/relationships; I experience too much stigma because of my work; I feel physically/mentally ill; I am retiring soon; Other reason, what?
What gender is your most typical client?	Man; Woman; Transman; Transwoman; Non-binary; Other, what?
What is your monthly gross income (before taxes) from providing sexual services?	0-499 €; 500-999 €; 1000-1499 €; 1500-1999 €; 2000-2499 €; 2500-2999 €; 3000-3499 €; 3500-3999 €; 4000-4499 €; 4500-4999 €; 5000-5499 €; 5500-5999 €; 6000-6499 €; 6500-6999 €; 7000-7499 €; 7500-7999 €; 8000-8499 €; 8500-8999 €; 9000-9499 €; 9500-9999 €; 10 000 € or more
What is your monthly gross income (before taxes) in total (from both providing sexual services and other work)?	0-499 €; 500-999 €; 1000-1499 €; 1500-1999 €; 2000-2499 €; 2500-2999 €; 3000-3499 €; 3500-3999 €; 4000-4499 €; 4500-4999 €; 5000-5499 €; 5500-5999 €; 6000-6499 €; 6500-6999 €; 7000-7499 €; 7500-7999 €; 8000-8499 €; 8500-8999 €; 9000-9499 €; 9500-9999 €; 10 000 € or more
Assess your economic situation over the last 6 months:	My economic situation is good, and I can save some of my income; My economic situation is good, but I spend all of my income; My

		economic situation is quite tight, and the money is just enough for the necessary expenses; My economic situation is tight and there is not enough money even for the necessary expenses; My money does not cover all the expenses and I had to take a loan
Modified AUDIT	*Have you used alcohol during the last 6 months? Also include those times when you drink only small amounts, such as a medium bottle of beer or a little bit of wine.<br>During the past 6 months...	Yes; No
	How often have you found that you were not able to stop drinking once you had started?	Never; Less than monthly; Monthly; Weekly; Daily or almost daily
	How often have you failed to do what was normally expected of you because of drinking?	Never; Less than monthly; Monthly; Weekly; Daily or almost daily
	How often have you needed a drink in the morning to get yourself going after a heavy drinking session?	Never; Less than monthly; Monthly; Weekly; Daily or almost daily
	How often have you had a feeling of guilt or remorse after drinking?	Never; Less than monthly; Monthly; Weekly; Daily or almost daily
	Have you been unable to remember what happened the night before because you had been drinking?	Never; Less than monthly; Monthly; Weekly; Daily or almost daily
	Have you or someone else been injured as a result of your drinking?	No; Yes, but not in the past 6 months; Yes, during the past 6 months
	Has a relative or friend, a doctor or another health worker been concerned about your drinking or suggested you cut down?	No; Yes, but not in the past 6 months; Yes, during the past 6 months
Modified DUDIT	*Have you used drugs during the last 6 months? Note. medicines are NOT considered drugs if they have been prescribed for you by a doctor and you are taking them in the doses prescribed by your doctor.	Yes; No
	Over the past 6 months, have you felt that your longing for drugs was so strong that you could not resist it?	Never; Less than monthly; Monthly; Weekly; Daily or almost daily
	Has it happened, over the past 6 months, that you have not been able to stop taking drugs once you started?	Never; Less than monthly; Monthly; Weekly; Daily or almost daily
	How often over the past 6 months have you taken drugs and then neglected to do something you should have done?	Never; Less than monthly; Monthly; Weekly; Daily or almost daily

How often over the past 6 months have you needed to take a drug the morning after heavy drug use the day before?	Never; Less than monthly; Monthly; Weekly; Daily or almost daily
How often over the past 6 months have you had guilt feelings or a bad conscience because you used drugs?	Never; Less than monthly; Monthly; Weekly; Daily or almost daily
Have you or anyone else been hurt (mentally or physically) because you used drugs?	No; Yes, but not in the past 6 months; Yes, during the past 6 months
Has a relative or a friend, a doctor or a nurse, or anyone else, been worried about your drug use or said to you that you should stop using drugs?	No; Yes, but not in the past 6 months; Yes, during the past 6 months

Note. Items marked with * were self-made questions added to a scale
